# Connexin 43 and Connexin 26 Involvement in the Ponatinib-Induced Cardiomyopathy: Sex-Related Differences in a Murine Model

**DOI:** 10.3390/ijms22115815

**Published:** 2021-05-28

**Authors:** Rosalinda Madonna, Stefania Moscato, Enza Polizzi, Damiana Pieragostino, Maria Concetta Cufaro, Piero Del Boccio, Francesco Bianchi, Raffaele De Caterina, Letizia Mattii

**Affiliations:** 1 Cardiology Division and Chair of Cardiology, University of Pisa, 56124 Pisa, Italy; rosalinda.madonna@unipi.it (R.M.); raffaele.decaterina@unipi.it (R.D.C.); 2 Department of Clinical and Experimental Medicine, University of Pisa, 56126 Pisa, Italy; stefania.moscato@unipi.it (S.M.); enza.polizzi@unipi.it (E.P.); francesco.bianchi@unipi.it (F.B.); 3 Department of Innovative Technologies in Medicine & Dentistry, University “G. d’Annunzio” of Chieti-Pescara, 66100 Chieti, Italy; dpieragostino@unich.it; 4 Center for Advanced Studies and Technology (CAST), University “G. d’Annunzio” of Chieti-Pescara, 66100 Chieti, Italy; maria.cufaro@unich.it (M.C.C.); piero.delboccio@unich.it (P.D.B.); 5 Department of Pharmacy, University “G. D’Annunzio” Chieti-Pescara, 66100 Chieti, Italy

**Keywords:** connexin, heart, mouse model, ponatinib, Notch1, sex-dependent differences, cardiotoxicity

## Abstract

Cardiac connexins (Cxs) are proteins responsible for proper heart function. They form gap junctions that mediate electrical and chemical signalling throughout the cardiac system, and thus enable a synchronized contraction. Connexins can also individually participate in many signal transduction pathways, interacting with intracellular proteins at various cellular compartments. Altered connexin expression and localization have been described in diseased myocardium and the aim of this study is to assess the involvement of Cx43, Cx26, and some related molecules in ponatinib-induced cardiac toxicity. Ponatinib is a new multi-tyrosine kinase inhibitor that has been successfully used against human malignancies, but its cardiotoxicity remains worrisome. Therefore, understanding its signaling mechanism is important to adopt potential anti cardiac damage strategies. Our experiments were performed on hearts from male and female mice treated with ponatinib and with ponatinib plus siRNA-Notch1 by using immunofluorescence, Western blotting, and proteomic analyses. The altered cardiac function and the change in Cxs expression observed in mice after ponatinib treatment, were results dependent on the Notch1 pathway and sex. Females showed a lower susceptibility to ponatinib than males. The downmodulation of cardiac Cx43, Cx26 and miR-122, high pS368-Cx43 phosphorylation, cell viability and survival activation could represent some of the female adaptative/compensatory reactions to ponatinib cardiotoxicity.

## 1. Introduction

Connexins (Cxs) are transmembrane proteins expressed on almost all mammalian cells. In humans there are 21 connexins named according to their molecular weight that range between 26 and 60 kDa. They are involved in cell-cell and cell-extracellular medium communication, allowing the exchange of small bioactive molecules such as ions, nucleotides, amino acids, sugars, intracellular mediators, and miRNAs. They play such a function by forming canals which are the hemichannels and the gap junction’s channels. Moreover, connexins can participate in various signal transduction pathways that interact individually with intracellular proteins [1,2,3].

The most expressed connexins in the normal adult heart are Cx26, Cx40, Cx43 and Cx45 [4]. The last three Cxs are mainly involved in forming gap junction structures and are generally located at the intercalated discs, allowing the propagation of electrical activity throughout the heart. This is one of the major reasons why their dysfunction has been linked to a wide variety of cardiac diseases [5]. In particular, the isotype Cx43 is the most studied and widely expressed as well as the most represented in cardiac myocyte gap junctions [6]. Cx26 expression has recently been found in mammalian cardiomyocytes by our group and, unlike the other cardiac connexins, it is distributed not in the intercalated discs but in the cytoplasm at the level of mitochondria, myofibrils, and cytoplasmic vesicles [7]. We demonstrated that the cardiac Cx26 protein is modulated early during aging [8] but until now, nothing is known about its function or its behaviour in cardiac diseases.

Tyrosine kinases inhibitors (TKIs) are drugs capable of specifically targeting tyrosine kinases receptors that frequently become abnormally hyper-activated in several human cancers. Ponatinib (Iclusig^TM^, Wilmignton, DE, USA) is a new orally active multi-tyrosine kinase inhibitor currently approved for patients with chronic myeloid leukaemia (CML) and Philadelphia chromosome-positive acute lymphoblastic leukaemia. It specifically targets native and mutated BCR-ABL tyrosine kinases. Moreover, a broader potential of ponatinib in the treatment of tumor diseases can be considered, since its inhibiting action has been demonstrated on other tyrosine kinases in other human malignancies also [9].

However, despite the impressive power of ponatinib in inhibiting various cancers, the drug is associated with vascular and cardiac toxicity with evidence of congestive heart failure and electrocardiographic abnormalities [10]. Several mechanisms have been hypothesized to explain the ponatinib-induced toxicity profile, i.e., the simultaneous inhibition of cardiovascular-related kinases [11], but further studies are needed to fully characterize the ponatinib signaling pathway. It is of interest to understand why the extent of the ponatinib-induced cardiotoxicity observed among the patients is uneven [12], and whether it is sex-related.

The Notch1 signaling influences multiple cell processes including differentiation, proliferation, apoptosis, migration, and angiogenesis. Notch1 has been shown to have both tumor suppressive and tumorigenic functions in different contexts [12]. Interestingly, it has been recently shown that a selective blockade of Notch1 can prevent vascular toxicity induced by ponatinib in human aortic endothelial cells [13]. Therefore, ponatinib treatment could specifically activate Notch signaling on tumour cells and this might represent the “on-target effect” on the tumor.

Against this background, the aim of the present study was to further explore the signaling pathway of ponatinib in a murine model of ponatinib-induced cardiotoxicity, focusing our interest on the connexins. We assessed the involvement of Cx43, Cx26, and some related molecules in ponatinib-induced cardiac toxicity especially considering the Notch1 signaling pathway and sex-related differences.

## 2. Results

### 2.1. Cardiac Function Analysis

Cardiac function was assessed by trans-thoracic echocardiography, while ponatinib-induced cardiac damage was assessed by cTn assay. The results, summarized in Table 1, showed the onset of a cardiac dysfunction in mice after treatment with ponatinib.

Treatment with ponatinib resulted in a 1.4- and 2.4-fold reduction in systolic function in females and males, respectively, as evidenced by the significant decrease in the left ventricular ejection fraction (LVEF) (*p* = 0.0196 in females and *p* = 9.412 × 10^−10^ in males). Sex-related differences, analysed by comparing male and female Δ_CNTRL-PON_, were statistically significant (15.36 ± 6 vs. 37.52 ± 2.32, respectively; *p* = 0.01). Treatment with PON + siRNA-Notch1 significantly reversed these changes both in females and males (*p* = 0.002 and *p* = 5.668 × 10^−6^, respectively).

Treatment with ponatinib did not affect diastolic function in both female and male mice, as demonstrated by the lack of significant differences between the PON-E/A ratio and the CNTRL-E/A ratio. However, the diastolic dysfunction was more accentuated in female controls than in their male counterparts, as represented by the lower female E/A ratio (*p* = 1.477 × 10^−9^).

A cardiotoxic effect of ponatinib was also demonstrated by the increased cTn levels found both in female and male mice (*p* = 0.001). The ponatinib-induced cTn increase was greater in males than in females (*p* = 0.03; Δ_CNTRL-PON_ = 0.029 ± 0.015 in males and 0.015 ± 0.007 in females. Treatment with siRNA-Notch1 prevented ponatinib cardiotoxicity both in females and males (*p* = 0.03).

### 2.2. Cx43 Protein and Related Partner Proteins Analysis

**Figure 1:**
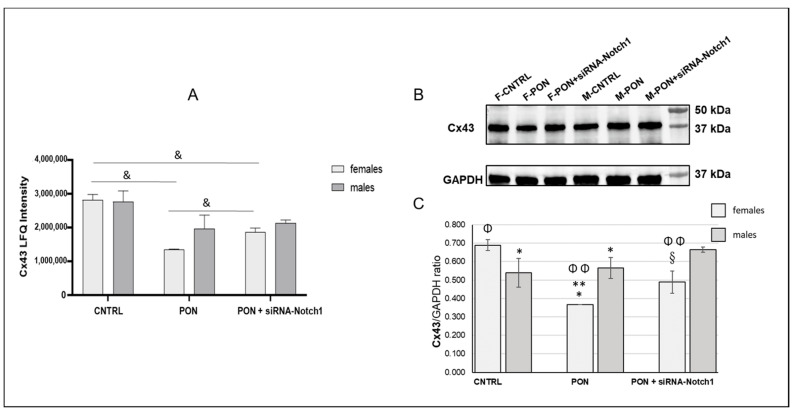
Heart Cx43 protein modulation. (**A**) Differential Proteomics Analysis demonstrated the downregulation of Cx43 only in female ponatinib-treated mice compared to control and siRNA-Notch1 ones. Histogram representing the proteomics data related to Cx43 abundance in heart mice samples. Data were reported as mean ± standard deviation of LFQ Intensity parameter analytical replicates (*n* = 3). &: *p* < 0.05 at ANOVA test and validated with post hoc-Tukey’s HSD test. Proteomics data were confirmed by Western Blot analysis as shown in Panel (**B**) of the figure. This shows a representative blot of one group of the three groups analysed. (**C**) Histogram representing the Cx43 protein expression in heart mice samples. Data are expressed as mean ± standard deviation, *n* = 3. ** *p* < 0.001 vs. CNTRL; * *p* < 0.05 vs. PON + siRNA-Notch1; ^§^: *p* < 0.01 vs. CNTRL; Φ: *p* < 0.05 vs. males; Φ Φ: *p* < 0.01 vs. males.

A quantitative proteomic analysis of the protein cargo of the hearts was carried out by using MaxQuant software and the individual protein expression was quantified by the LFQ Intensity parameter. We quantified 476 proteins in control female hearts, 847 proteins in ponatinib-treated female hearts, and 856 proteins in PON + siRNA-Notch1-treated female hearts. At the same time, 657 proteins were quantified in control male hearts, 803 proteins in ponatinib-treated male hearts, and 800 proteins in PON + siRNA-Notch1-treated male hearts. To identify differential expression proteins, we performed a statistical comparison analysis for each sex-related group. The expression levels of all the proteins were measured as an analytical triplicate average for each different treatment for both female and male groups. In particular, significantly expressed proteins were determined by the ANOVA test (*p*-value < 0.05) and then validated with post-hoc Tukey’s HSD test, which obtained 452 and 334 significantly differential proteins between three female and three male mice hearts, respectively. The whole list of differential proteins is reported in Supplementary Tables S1 and S2. Our proteomics analysis identified Cx43 as a significantly down-expressed protein only in female ponatinib groups compared to control and siRNA-Notch1 groups, while in the male group the Cx43 levels were unchanged (Figure 1A).

Other molecules were differently regulated in female and male mice hearts by ponatinib treatment, and, among these, our focus was on those related to Cx43. Namely, signal-regulated kinase (ERK), kinase B (Akt), microRNA122 (miR-122), and interleukin-6 (IL-6) were analyzed [14,15,16,17].

**Figure 2:**
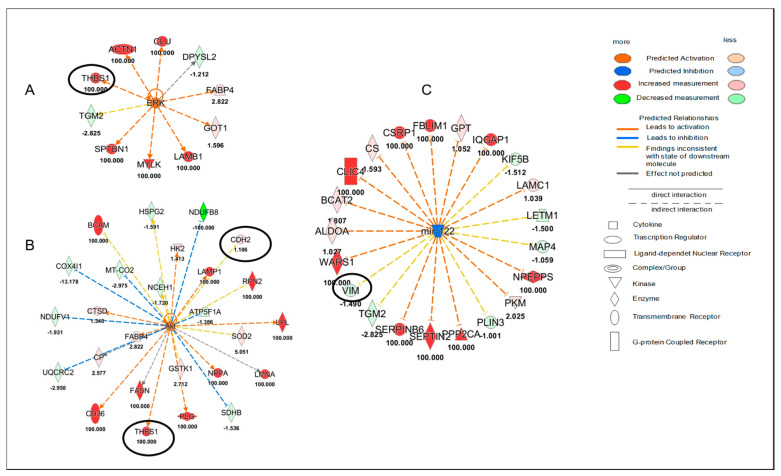
Female proteomic analysis. Upstream network effects of a quantified protein in the Ingenuity Pathway Analysis (IPA) revealed the activation of ERK (**A**) and Akt (**B**) as well as the inhibition of miR-122 (**C**) in female mice cardiac tissue treated with ponatinib compared to control ones. Red and green shapes represent increased or decreased measurements of quantified proteins, respectively. Color intensity is directly proportional to the fold change value of each protein reported in the figure. Circles show proteins related to Cx43. Color key and symbols are reported in the legend.

In this regard, quantitative proteomics data were used for a functional enrichment analysis. The protein ratio was used for “Core Analysis” through the Ingenuity Pathway Analysis (IPA software) to compare the heart tissue of the ponatinib groups (both females and males) with controls and siRNA-Notch1 ones. In particular, Upstream Regulator Analysis (URA) highlighted that ERK (*z*-score = 2.36, *p*-value = 0.045) and Akt (*z*-score = 2.00, *p*-value = 3.62 × 10^−3^) were significantly up-regulated after ponatinib treatment in females (Figure 2A,B), while the miR-122 (*z*-score = −3.38, *p*-value = 3.38 × 10^−11^) was down-regulated compared to control ones (Figure 2C).

**Figure 3:**
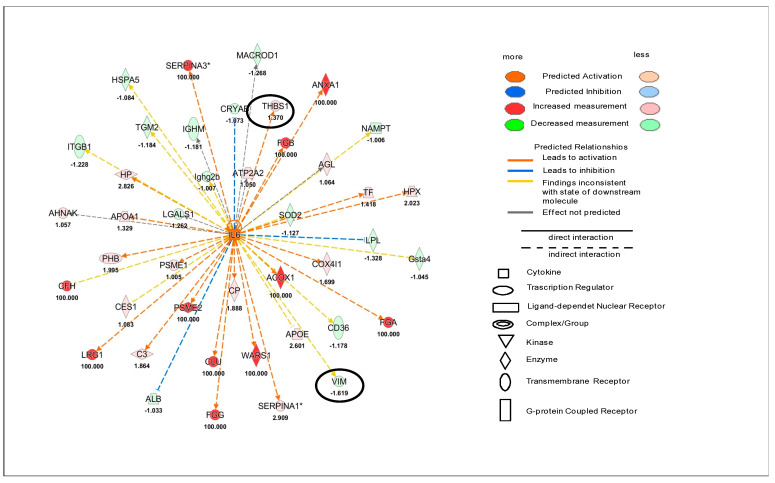
Male proteomic analysis. Upstream network effects of a quantified protein in the Ingenuity Pathway Analysis (IPA) revealed the activation of IL6 in male mice cardiac tissue treated with ponatinib compared to control ones. Red and green shapes represent increased or decreased measurements of identified proteins, respectively. Color intensity is directly proportional to the fold change value of each protein reported in the figure. Circles show target molecules of Cx43. Color key and symbols are reported in the legend.

In the male groups, IL-6 was significantly up-regulated (*z*-score = 2.52, *p*-value = 8.8 × 10^−3^) in males treated with ponatinib compared to control ones (Figure 3). Interestingly, the upstream networks show how quantified proteins involved in the regulation of ERK, Akt, miR-122, and IL-6 included one or two proteins which were identified as Cx43 target molecules in the dataset. These proteins were *N*-cadherin (CDH2), thrombospondin 1 (THBS1), and vimentin (VIM) (networks in Figure 1 and Figure 2).

Sex-related differences were also found in the activated and inhibited diseases and biofunctions in ponatinib treated female and male hearts compared to controls. The main Cx43-dependent processes are listed in Table 2. In detail, the orange and the blue color indicate the predicted activation (*z*-score ≥ 2.00) and inhibition (*z*-score ≤ −2.00), respectively. As we can see, the protein cargo of female hearts treated with ponatinib was able to trigger some processes involving Cx43, such as the activation of cell viability, cell survival, and molecule transport as well as the inhibition of organismal death. On the contrary, the protein cargo of male hearts treated with ponatinib did not significantly trigger any processes involving Cx43. It was observed that the triggering by ponatinib treatment of the above-mentioned processes was overturned by siRNA-Notch1 treatment (data not shown).

Western blotting performed for Cx43 on heart protein samples confirmed the proteomic data. Indeed, we observed a significant decrease in the Cx43 protein in the heart samples of females treated with ponatinib, compared to controls and the siRNA-Notch1 treated samples.

However, the siRNA-Notch1 treatment did not completely restore Cx43 amount in control female hearts. No significant change in Cx43 protein expression was observed in the male heart samples from both ponatinib- and siRNA-Notch1-treated mice. The amount of Cx43 in control hearts was higher in females than in males (Figure 1B,C), which was different from the results from the proteomic analysis. 

**Figure 4:**
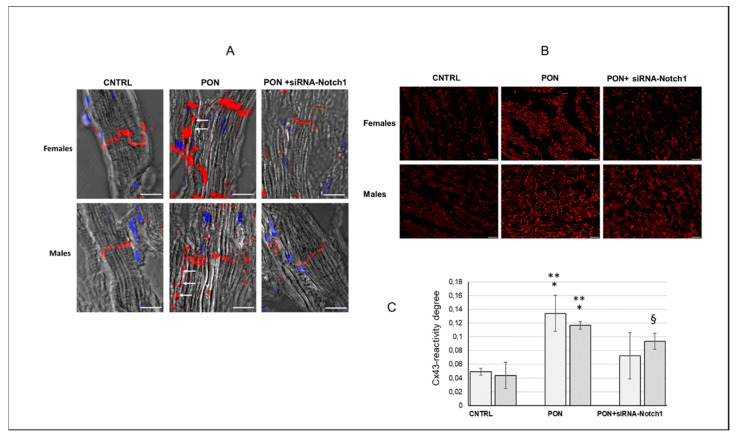
Cx43 protein expression at cardiomyocyte membrane level. (**A**) Confocal laser scanning microscopy: representative three-dimensional images of the maximum intensity projection of mice cardiomyocyte longitudinal sections. Cx43 (red) is confined at the intercalated discs in CNTRL and in siRNA-Notch1 groups whereas it lies also on the lateral border (arrows) of cardiomyocytes (grey) in PON groups. Nuclei of cardiomyocytes and connective cells are marked with DAPI in blue. Magnification: 600×. Bars: 10 µm. (**B**) Immunofluorescence staining: representative images of Cx43 immunoreaction (red). Magnification: 200×. Bars: 50 µm. (**C**) Image analysis of Cx43-immunofluorescence on cardiomyocytes. Data are expressed as mean ± standard deviation, *n* = 4. * *p* < 0.001 vs. CNTRL; ** *p* < 0.05 vs. PON + siRNA-Notch1; ^§^: *p* < 0.001 vs. CNTRL.

Cx43 is widely expressed in cardiac tissue present in cardiomyocytes but is also present in stroma cells and vessels. Therefore, the use of optical microscopy and immunohistochemistry allowed us to analyse the Cx43 protein specifically localized at the level of cardiomyocytes as well as at the level of membrane cardiomyocytes. Namely, in the female and male control groups, Cx43 was mainly confined to the intercalated discs area. However, in mice treated with ponatinib, a tangible amount of Cx43 was present also along the lateral borders of the cardiomyocytes. Cx43 lateralization was avoided by siRNA-Notch1 treatment (Figure 4A,B). Interestingly and differently from the data obtained by proteomic analysis and Western blotting, ponatinib treatment increased the membrane Cx43 both in female and male cardiomyocytes on a significant level compared to control and siRNA-Notch1 treatments. The amount of membrane Cx43 tended to be higher in females than in their male counterparts in the control and ponatinib treated groups, however not in a significant way. Only in the female groups did the siRNA-Notch1 treatment completely revert ponatinib action (image analysis in Figure 4C).

As the specific permeability of Cx43-made junctions is regulated, at least in part, through protein kinase C (PKC)-dependent phosphorylation of Cx43 at serine 368 (S368), we tested the protein expression of Cx43 phosphorylated at S368 (pS368-Cx43) and the activity of PKC. Specifically, we indirectly studied PKC activity, detecting the levels of PKC phosphorylated substrates (PKCps) in the cardiomyocytes.

**Figure 5:**
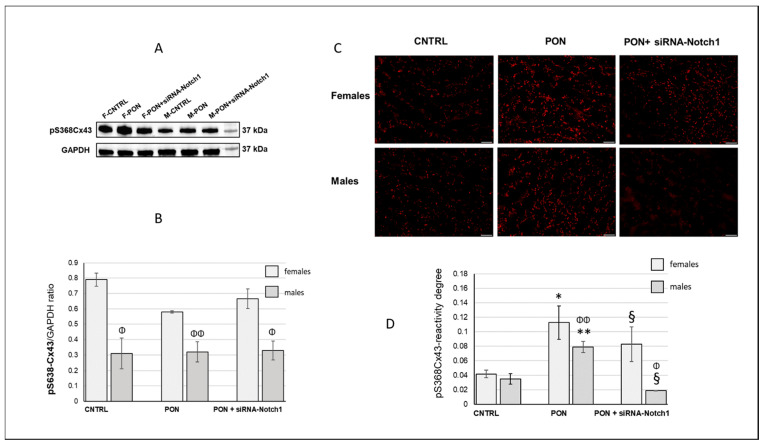
pS368-Cx43 protein expression. (**A**) Representative of a Western blot analysis of one group of the three analysed groups. (**B**) Histogram obtained by Western blot analysis, representing the total cardiac pS368-Cx43 protein expression of heart mice samples. (**C**) Immunofluorescence staining: representative images of cardiomyocyte pS368-Cx43 immunoreaction (red). Magnification: 200×. Bars: 50 µm. (**D**) Image analysis of cardiomyocyte pS368Cx43-immunofluorescence. Data are expressed as mean ± standard deviation, *n* = 4. * *p* < 0.001 vs. CNTRL; ** *p* ≤ 0.001 vs. CNTRL and PON + siRNA-Notch1; ^§^: *p* ≤ 0.001 vs. CNTRL; Φ: *p* < 0.01 vs. females; Φ Φ: *p* < 0.05 vs. females.

Western blotting analysis revealed a higher amount of cardiac pS368-Cx43 in females than in males, but no differences were found among controls and the treated groups, both for females and males (Figure 5A,B). Conversely, by immunofluorescence observation we detected pS368-Cx43 immunopositivity at low levels in control cardiomyocytes but at high levels in ponatinib treated ones. Most of pS368-Cx43 was localized at the intercalated discs and, in ponatinib treated groups, also at the lateral borders. In the siRNA-Notch1 treated samples, the immunoreaction in males was lower than in both ponatinib treated ones and controls, whereas in females the pS368-Cx43 staining was the same as in ponatinib treated groups (Figure 5C). Image analysis confirmed the pS368-Cx43 protein modulations detected by microscopic observation, demonstrating a greater significant amount of pS368-Cx43 in ponatinib treated mice. Moreover, siRNA-Notch1 treatment further reduced the pS368-Cx43 immunoreaction compared to controls in females. Interestingly, the female cardiomyocytes exhibited a higher amount of pS638-Cx43 than the male ones in the controls as well as the groups treated with ponatinib and siRNA-Notch1 (Figure 5B).

**Figure 6:**
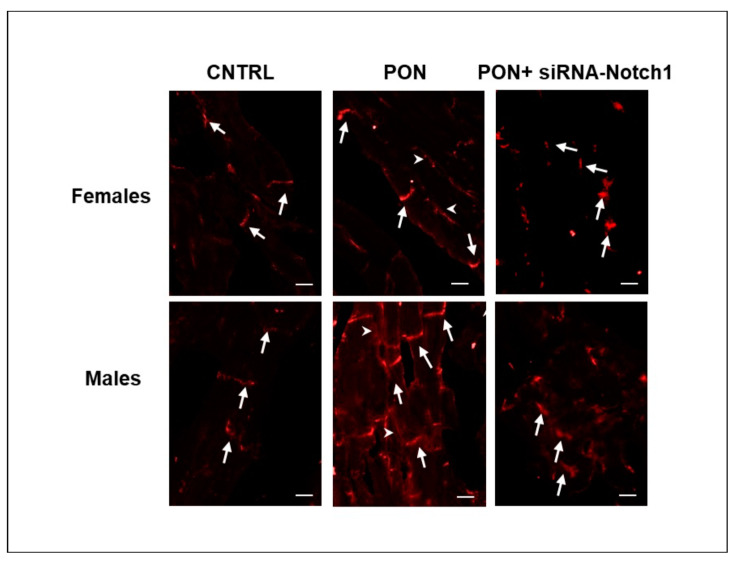
PKCps immunofluorescence staining in cardiomyocytes. Representative images of PKCps immunopositivity (red) at intercalated discs (arrows) and at lateral borders (arrowheads). Magnification: 200×. Bars: 10 µm.

The results obtained by immunofluorescence observation showed the presence of PKCps at level of intercalated discs in control groups. In ponatinib treated groups, PKCps were found at the level of both the intercalated discs and the lateral borders of cardiomyocytes. The immunopositivity of PKCps appeared higher, especially for males, in ponatinib treated groups than in controls. In siRNA-Notch1 treated groups, PKCps were expressed at the intercalated discs and with apparently less intensity than in the ponatinib counterparts (Figure 6).

### 2.3. Cx26 Protein Analysis

**Figure 7:**
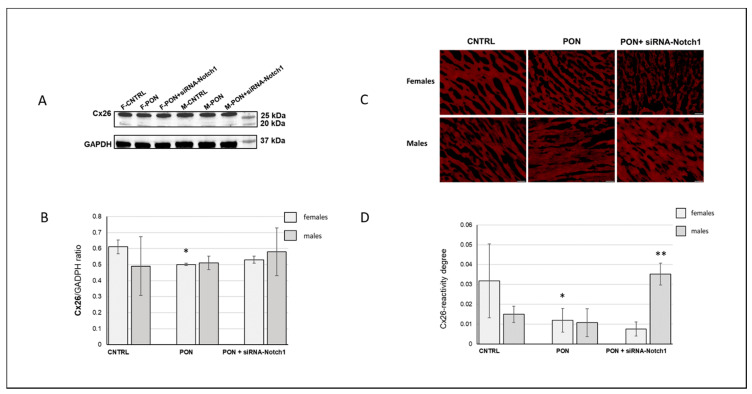
Cardiac Cx26 protein expression. (**A**) Representative of a Western blot analysis of one group of the three analysed groups. (**B**) Histogram obtained by Western blot analysis, representing the total cardiac Cx26 protein expression of heart mice samples. (**C**) Immunofluorescence staining: representative images of cardiomyocyte Cx26 immunoreaction (red). Magnification: 200×. Bars: 50 µm. (**D**) Image analysis of cardiomyocyte Cx26 immunostaining * *p* ≤ 0.05 vs. CNTRL and PON + siRNA-Notch1; ** *p* < 0.01 vs. CNTRL and PON + siRNA-Notch1.

Western blotting for Cx26 showed a significantly decreased amount of the protein after ponatinib treatment than in controls, but only in female hearts. Cx26 expression in control groups was higher in females than in males but not at a significant level (Figure 7A,B).

Cx26 cardiomyocyte immunopositivity was detected by immunofluorescence microscopy distributed throughout the cell, as we had expected. Ponatinib induced a decrease in Cx26 in female cardiomyocytes only. Moreover, siRNA-Notch1 cotreatment ulteriorly reduced Cx26 in female cardiomyocytes while it increased Cx26 in male cardiomyocytes with respect to control and the ponatinib treated groups (Figure 7C,D).

## 3. Discussion

Among cardiac Cxs, Cx26 and Cx43 are the ones that are widely expressed throughout the heart. Cx26 is present in vessels, as well as in working and conducting cardiomyocytes, and its localization is scattered all over the cell [7]. Having been found in cardiomyocytes only recently, its role in cardiac function and disease remains poorly understood. Cx43 is expressed in vessels, stroma cells and working cardiomyocytes. In cardiomyocytes, it is mainly localized at the level of the intercalated discs where it forms gap junctions and, carrying on its canonical function, it allows the propagation of electrical activity throughout the heart. Moreover, it can form hemichannels at intercalated discs, at membrane lateral borders and at mitochondrial membranes. Hemichannels regulate extracellular communications, especially allowing the dissemination of disease. Cx43 is also involved in various signalling transduction pathways that interact individually with intracellular proteins [1,2,3,18,19]. Cx43 can undergo some posttranslational modifications, including phosphorylation. Its phosphorylation, which can occur at various specific serine residues, regulates channel assembly/disassembly, localization, and gap junction/hemichannel permeability. Consequently, this regulation will affect cardiac disease development. Several protein kinases are implicated in Cx43 phosphorylation, such as PKC, ERK, and Akt [14]. Cx43 phosphorylation by ERK and PKC is typically increased during gap junction remodelling and disassembly while Akt activity increases with the gap junction size. Together, they can protect the myocardium from ischemia reperfusion injury. ERK and Akt both phosphorylate Cx43, and, at the same time, they can be controlled themselves by the phosphorylation status of Cx43, in response to ischemic injury. It has been demonstrated that a decreased Cx43 phosphorylation at serine 368 increases arrhythmia occurrence in cultured cardiac myocytes and in vivo in animal models. However, the outcome of Cx43 phosphorylation depends on the type of the kinase, the cell target, and the cellular context [19].

In the present study, we evaluated the modulation of Cx43, Cx26 and some of their related partners in female and male hearts of a murine model of cardiotoxicity. In particular, we explored a possible Notch1/Cxs signaling pathway triggered by ponatinib, a new multi-tyrosine kinase inhibitor that has been successfully used against human malignancies which, however, causes cardiac toxicity. The results that have been obtained can be summarized as follows. Firstly, analyses of the cardiac function showed a different sex-related susceptibility to ponatinib treatment, higher in males than in females, as demonstrated by the greater cardiac dysfunctions in male rather than in female mice. Moreover, the observed revertant action of siRNA-Notch1 cotreatment evidenced that ponatinib exerts its cardiotoxic effects through the Notch1 pathway, both in females and males. In addition, regarding Cxs, we found that ponatinib influenced the protein expression of both Cx43 and Cx26 as well as related molecules, often through the Notch1 signaling pathway and in a sex-related mode. The results that have been obtained are interesting for their novelty, for their support of previous observations, and for future research inspirations.

More specifically, the results have demonstrated:

(1) A higher expression of cardiac Cx43, pS368-Cx43 and Cx26 in female mice rather than in male mice under basal conditions. Interestingly, the fact that there was a higher amount of Cx43 in female control groups than in their male counterparts is in agreement with previous studies and demonstrates that this sex-dependent difference in Cx43 expression under basal conditions was preserved during aging. Indeed, the mice in our study were very old and were older than the animals used in previous works [20,21]. On the other hand, the higher basal cardiac Cx26 expression in female mice compared to male mice has been demonstrated in the present study for the first time. In this regard, it would be interesting to investigate if the sex-dependent Cx expression can be extended to the other cardiac Cxs isotypes.

(2) The cardiotoxic effect of ponatinib influenced Cx43 and Cx26 expression, often in a sex-related mode. Indeed, in ponatinib treated groups compared to controls, Cx43 and its S368 phosphorylated form increased at the level of the membrane cardiomyocytes and exhibited a disordered distribution. Again, the increase in Cx43 and pS368Cx43 was higher in females than in males. An improved expression of Cx43 and its pS368-phosphorylated form as well as their disordered distribution has been found previously in myocarditis and it has been suggested that this could favour a cardioprotective phenotype, especially in females [20,21,22,23]. Ponatinib affected cardiomyocyte Cx26 expression differently as it induced its decrease, but only in females. However, the amounts of total cardiac and cardiomyocyte Cx26 were always higher in females than in males both for controls and for ponatinib treated groups. It has to be noted that the Cx26 expression was often highly variable in the samples from the same group, as shown by the large error bars (Figure 7B,D). Consequently, Cx26 expression could be influenced also by factors on a case-by-case basis. Therefore, further studies will be necessary to ascertain the real involvement of Cx26 in heart injury. Due to the poor literature data on cardiac Cx26, this result is very important as it demonstrates a modulation of Cx26 expression in the response to heart injury.

In conclusion, the lower susceptibility to ponatinib shown by females could be due, at least in part, to Cx regulation which appeared always present or more marked in females than males.

(3) The results on the amounts of Cx43 and pS368-Cx43, which were obtained from whole heart samples, were different from those obtained from the cardiomyocytes analysis performed by immunofluorescence microscopy. Indeed, total cardiac Cx43, in a different way from cardiomyocyte Cx43, decreased after ponatinib treatment in females while it did not change in males. The same was shown for total cardiac pS368-Cx43 that appeared to not be influenced by ponatinib treatment either in females or in males. Instead, total cardiac Cx26 decreased after ponatinib treatment, in a similar way to cardiomyocytes Cx26 in females. The differences between total cardiac and cardiomyocyte Cx43 expression as well as those between female and male Cx43 modulation could be due to the presence of this connexin in vessels and stromal cells too. Thus, ponatinib could influence Cx43 both by reducing its expression in cardiac stroma cells and enhancing its expression in cardiomyocyte membranes, however in a more marked manner in females. The same can happen to pS368-Cx43, as ponatinib could stimulate Cx43 phosphorylation mainly at the level of intercalated discs and to a minor extent at the level of vessels and stroma cells, compared to controls, and always in more marked way for females. The observed increase in PKC activity at the intercalated discs in the cardiomyocytes of mice treated with ponatinib compared to controls could corroborate this assumption. However, to better characterize this interpretation, further studies are needed to examine the specific Cx modulation also in cardiac vessels and stromal cells.

(4) Ponatinib action was sex-dependent concerning Akt, ERK and miR-122 molecules that were modulated after ponatinib treatment but only in female hearts. Akt and ERK were upregulated, while miR-122 was downregulated. This miRNA appears to be directly implicated in the development of cardiovascular diseases and its inhibition plays antifibrotic, anti-apoptotic, anti-inflammatory, and antioxidant functions [16]. MiR-122 could be linked to Cxs for their own involvement in miRNA intercellular transfer [15]. Thus, we can speculate that the observed reduction in cardiac Cx43 and Cx26 in female hearts could be, in part, responsible for miR-122 down regulation. Akt and ERK could regulate Cx43 phosphorylation and consequently modulate its expression in an independent way at the level of various heart components. The proteomic analysis revealed that the regulation of Akt, ERK and miR-122 indirectly involved Cx43. Indeed, among the several proteins regulating them, there were VIM, CDH2 and THBS1, which were identified as Cx43 target molecules. However, THBS1 was only significantly upregulated and this happened in females only. Interestingly, it has been suggested that an increase in THBS signaling in the heart during stress and haemodynamic overload might be beneficial [24].

In addition to the above-described potential cardio protective mechanisms, female mice also showed an activation of Cx43-related cell functions at the cardiac level, such as cell viability and survival, as well as the transport of molecules, which could be consistent with an adaptative/compensatory reaction to ponatinib cardiotoxic action.

On the other hand, male hearts did not present the activation or inactivation of specific Cx43-related cell functions after ponatinib treatment. Il-6 up regulation was found at cardiac level only in male mice. This condition could be involved in the higher susceptibility to ponatinib treatment of males compared to females. Indeed, it has been demonstrated that cardiac long-term IL-6 signalling or cardiac over production of IL-6R have a causal role in cardiovascular disease [25].

(5) Silencing the Notch1 signaling obtained by using siRNA-Notch1 treatment allowed us to demonstrate that the Notch1/Cxs pathway is involved in the cardiotoxic effect of ponatinib. Indeed, a ponatinib-induced modulation of Cx43 expression and phosphorylation as well as some specific cell functions were prevented by siRNA-Notch1 cotreatment. However, silencing Notch1 did not reverse Cx26 modulation, total cardiac Cx43 in males, pS368-Cx43, Akt, ERK, miR122, and IL-6 modulation, suggesting that Notch1 is not the only mediator of the ponatinib effect on Cxs regulation. On the other hand, cardiac dysfunctions observed in mice treated with ponatinib were prevented by siRNA-Notch1 treatment confirming the involvement of the other molecules (Table S1), in addition to Cxs, in ponatinib/Notch1 signaling pathway.

## 4. Materials and Methods

### 4.1. Materials, Animals and Treatments

Ponatinib (PON) was acquired by Incyte S.r.l (Wilmington, DE, USA). SiRNA-Notch1 and siRNA-scrambled were purchased from Invitrogen Life Technologies (Carlsbad, CA, USA).

Male and female C57BL/6 mice (body weight: 30 ± 4 g, 24 months-old) were purchased from Charles River Italia (Lecco, Italy). Mice were housed under a 12 h light/dark cycle in temperature and humidity-controlled rooms and were provided with ad libitum rodent chow (Teklad 7001, 4.4%; Harlan Teklad Global Diets, Madison, WI, USA) and water. Animals were randomized into 3 groups (*n* = 12, *n* = 6 male and *n* = 6 female for each treatment group): control (CNTRL), PON + siRNA-scrambled (PON), PON + siRNA-Notch1 (PON + siRNot). PON was dissolved in DMSO and administered to mice in the experimental groups by oral gavage daily (30 mg/kg/d for 28 days, corresponding to the oral doses clinically used in humans: https://go.drugbank.com/drugs/DB08901, accessed on 25 February 2019), with siRNA-Notch1 or siRNA-scrambled administered via tail vein every 3 days. The control group was given the same dosage of DMSO dissolved in the same volume of water for 28 days. Mice were then euthanized by deep anesthesia under 2% isoflurane. A terminal blood sample was immediately drawn from the left ventricle and later it was used for cardiac troponin (cTn) dosage. Hearts were excised, snap-frozen in liquid N_2_ and stored at −80 °C for protein extraction or embedded in an optical cutting temperature (OCT) medium and stored at −80 °C for immunohistological analyses. All procedures were approved by the local Institutional Ethics Committee for Animal Research (Protocol number 176/2019-R released in 25 February 2019). All studies complied with the Guidelines from Directive 2010/63 EU of the European Parliament on the protection of animals used for scientific purpose of the NIH guidelines.

Non-viral siRNA delivery in vivo was performed by tail vein injection. For monitoring the transfection efficacy, we used scrambled siRNA conjugated with a Cy3 fluorochrome provided by Mirus (Mirus Bio, Madison, WI, USA). Accordingly, 1.33 μL (25 nM) of Cy3-conjugated scrambled siRNA or Cy3-conjugated siRNA-Notch (NM_008714.3) were combined with 0.5 μL of Transit-TKO (Mirus) in a final volume 10 μL sterile H_2_O, and incubated for 30 min at room temperature, according to the protocol previously published [26]. According to mice randomization, tail vein injections of 10 μL phosphate-buffered saline (PBS) (*n* = 6 male mice and *n* = 6 female mice), or 10 μL siRNA-scrambled (*n* = 6 male mice and *n* = 6 female mice), or 10 μL siRNA-Notch1 (*n* = 6 male mice and *n* = 6 female mice) were performed with a 32 gauge needle attached to a 1 mL syringe (Beckton Dickinson, Franklin Lake, NJ, USA). Injections were repeated after 72 h and every three days from the first injection.

### 4.2. Echocardiography

Using a portable ultrasound apparatus (Esaote; Genoa, Italy) equipped with a 21-MHz linear probe, we performed transthoracic echocardiography 28 days after treatments to assess the functional effects of each treatment. The mice were anesthetized (ketamine, 100 mg/kg) and placed in the left lateral decubitus position. To evaluate LV structural changes, the following parameters were measured: LV end-diastolic diameter [LVEDD] and LV end-systolic diameter [LVESD; left ventricular fractional shortening (FS%), calculated as: FS (%) = ([LVEDD–LVESD)/LVEDD] × 100; left ventricular ejection fraction (EF%), calculated as an index of systolic function, ([LVEDD3–LVESD3)/LVEDD3] × 100. The same parameters were measured on control, non-manipulated healthy animals. Passive LV filling peak velocity (E, mm/s) and atrial contraction flow peak velocity (A, mm/s) were obtained by Pulsed-wave Doppler through the mitral valve. Each measurement was obtained by averaging the results of 3 consecutive heart beats. Individuals conducting the echocardiography were blinded to the animal treatment groups.

### 4.3. Label-Free Proteomics Analysis and Bioinformatics

Cardiac tissues from each treatment group were prepared according to the Filter Aided Sample Preparation (FASP) method. Briefly, cardiac tissues were lysed by sonication in a RIPA buffer and centrifuged to remove cell debris. A total of 50 µg of proteins for each treatment were digested by trypsin (Promega, Madison, WI, USA). For protein label free identification and quantification, tryptic peptides from each sample were analyzed in triplicate with LC-MS/MS using the UltiMateTM 3000 UPLC (Thermo Fisher Scientific, Waltham, MA, USA) chromatographic system coupled to the Orbitrap FusionTM TribridTM (Thermo Fisher Scientific) mass spectrometer. Peptides were loaded on the Trap Cartridge C18 (0.3 mm ID, 5 mm L, 5 μm PS, Thermo Fisher Scientific) and then separated on an EASY-spray AcclaimTM PepMapTM C18 (75 μm ID, 25 cm L, 2 μm PS, Thermo Fisher Scientific) nanoscale chromatographic column. Mobile phase A was 0.1% formic acid in H2O and mobile phase B was 0.1% formic acid in acetonitrile. The flow rate was set at 300 nL/min, with a total run time of 120 min using a chromatographic gradient from 2 to 90% of phase B. Proteomics data were acquired in positive-ion polarity with a Data Dependent Acquisition (DDA) mode to trigger precursor isolation and the MS2 sequence using N2 as a collision gas for CID fragmentation. MS1 scans were performed in the Orbitrap analyzer covering a *m/z* range of 375–1500 with 120,000 of resolution. The signal intensity threshold was set to 5 × 10^3^ and the MS2 spectra were acquired using a Top Speed method of 3 s. In particular, precursor ions with charges of +2 to +7 were used for MS2 sequencing and scanned in the ion trap by setting the following parameters: MS2 isolation window of 1.6 Da, AGC target of 2 × 10^3^, a dynamic exclusion time of 60 s and mass tolerance of ± 10 ppm were used. We performed a CID fragmentation with a fixed collision energy of 35% and an activation time of 10 ms.

Proteomics raw data were processed using a free computational platform, MaxQuant v1.6.6.0 (Max-Planck Institute for Biochemistry, Martinsried, Germany). Peak lists, generated in MaxQuant, were searched using the Andromeda peptide search engine against the UniProt database (released 2019_11, taxonomy Mus Musculus, 21,990 entries) supplemented with frequently observed contaminants and containing forward and reverse sequences. LFQ Intensity was used to quantify protein abundance in each sample. Bioinformatics analysis was performed with Perseus v1.6.10.50 (Max-Planck Institute for Biochemistry, Martinsried, Germany). LFQ intensities were log2 transformed to facilitate the calculation of the protein expression. The minimum number of valid values accepted was set at 2 in at least one group.

Finally, the protein ratio was uploaded for “Core Analysis” through the Ingenuity Pathway Analysis tool, (IPA, Qiagen, Hilden, Germany). IPA is able to statistically map the modulated proteins for their functional annotations, such as Canonical Pathways, Upstream Regulators Analysis and downstream effects networks, through Gene Ontology and pathway analysis. In this way, it is possible to identify the metabolic pathways and the secondary genes/proteins inhibited (*z*-score ≤ −2.00) and/or stimulated (*z*-score ≥ 2.00) for a specific phenotype and consequently classify potential effectors molecules and/or a pharmacological target.

### 4.4. Western Blotting

Cardiac tissue samples were obtained and homogenized in a RadioImmuno Precipitation Assay (RIPA) buffer containing a protease inhibitor cocktail 1× (S8830, Sigma Aldrich, Merck KGaA, Darmstadt, Germany) at 4 °C. After centrifugation at 15,000 rpm for 20 min at 4 °C, supernatants were collected and the protein concentration was evaluated by the bicinchoninic acid assay (BCA) (Pierce™, Thermo Fisher Scientific) microplate method.

Proteins (30 µg/lane) were separated under reducing conditions on a 4–20% polyacrylamide gel (BioRad, Hercules, CA, USA) and electroblotted onto a nitrocellulose membrane using the Trans Turbo Blot system (BioRad). After blocking in 5% dry fat milk (Sigma Aldrich) in Tris Buffered Saline solution containing 0.1% Tween 20 (T-TBS) for 1 h at room temperature (RT), the membranes were incubated overnight at 4 °C with the following antibodies diluted in T-TBS: (1) anti-Cx43; (2) anti pS368-Cx43; (3) anti-Cx26; (4) anti-GAPDH as protein loading control (see Table 3). Anti-Rabbit-HRP-conjugated secondary antibodies (BioRad), diluted in T-TBS containing 5% of dry fat milk, were used and the chemiluminescent signal (ECL clarity, BioRad) was detected using Chemi-Doc XR (BioRad). The intensity of the bands was measured using the Image Lab Software (BioRad) on *n* = 3 different series of samples.

### 4.5. Immunofluorescence Analysis

Immunofluorescence was performed on 5–8 μm thick sections from OCT-embedded hearts as previously described [8]. Briefly, the sections were treated with 0.2% triton-X100/PBS for antigen retrieval and successively with blocking solution (BS, 0.1% Tween, 0.25% BSA in PBS) for 1 h at room temperature (RT). Then, the samples were incubated overnight at 4 °C with previously tested primary antibodies [7,19,27] diluted in BS (see Table 3).

After washing in BS, sections were incubated for 90 min at RT in the dark with fluorescent anti-rabbit secondary antibody (see Table 3). Samples on slides were finally mounted with a mounting medium (Prolong^TM^ Diamond Antifade Mountant with DAPI, Thermo Fisher Scientific) and observed under a fluorescence microscope (BX43, Olympus, Hamburg, Germany). Images were captured at 200× total magnification (20× objective magnification and 10× ocular magnification) by a SC50 digital camera (Olympus; pixel dimension photocamera sensor 2.2 × 2.2 μm, LED light). Cx43 immunofluorescence images were also captured by a confocal laser scanning microscopy (TC SSP8 Leica Microsystems, Mannheim, Germany) using 40× oil immersion lenses by with 488 nm and 561 nm excitation wavelength lasers. Negative controls for secondary antibodies were performed by omitting primary antibodies and incubating the specimens with nonimmune serum or BS.

### 4.6. Image Analysis

Immunofluorescence positivity was quantified by image analysis on cardiomyocytes from female and male mice hearts. Three nonconsecutive sections were examined per sample and the microscopic fields were objectively selected in order to cover the whole section. Moreover, in every microscopic field of the sections, tissue area containing only cardiomyocytes was selected. The cardiomyocyte reacting areas were quantified with CellSens Imaging Software (Olympus).

The medium colour threshold (the level above which the pictures were considered to be reacting) was evaluated for every sample on negative controls. The image analysis was performed by considering the percentage of the reacting area and the respective level of pixel colour intensity per field. The final rate of reactivity degree of each group was calculated as the product between the average of the positive area percentage and the mean value of the pixel color intensity per microscopic field.

### 4.7. Statistical Analysis

Data are expressed as mean ± standard deviation (SD). Two group comparisons were performed by using the Student’s *t*-test for unpaired values. Multiple test correction was completed using the ANOVA test with a *p*-value threshold of 0.05 and Tukey’s HSD test was used as post-hoc test to determine statistical significance within and between groups.

## 5. Conclusions

We demonstrated a sex-dependent ponatinib cardiotoxicity and a sex-related Cxs involvement in C57BL/6 mice. Moreover, we identified a possible Notch1/Cxs signaling pathway for sex-dependent ponatinib cardiotoxicity and as the consequence, the possibility to improve the effectiveness of ponatinib clinical therapy, interfering with this pathway.

## Figures and Tables

**Table 1:** Cardiac function data.

**(a) Echocardiographic Data**						
	**LVEDD (mm)**			**LVESD (mm)**		
	**CNTRL**	**PON**	**PON+siRNA-Not1**	**CNTRL**	**PON**	**PON+siRNA-Not1**
**Females**	2.74 ± 0.61	4.53 ± 0.2	3.94 ± 0.4	1.23 ± 0.58	2.9 ± 0.22	1.89 ± 0.47
**Males**	4.34 ± 0.61	6.11 ± 0.52	4.27 ± 0.60	1.48 ± 0.27	4.37 ± 0.55	2.01 ± 0.58
	**LVEF (%)**			**[E] wave (cm/sec)**		
	**CNTRL**	**PON**	**PON+siRNA-Not1**	**CNTRL**	**PON**	**PON+siRNA-Not1**
**Females**	51.5 ± 13.51	36.14 ± 7.54	52.14 ± 7.92	21.65 ± 11.37	29.53 ± 11.63	28.18 ± 10.18
**Males**	65.3 ± 6.43	27.73 ± 8.75	53.1 ± 9.95	45.02 ± 5.58	60.55 ± 16.21	48.57 ± 8.50
	**[A] wave (cm/sec)**			**[E/A] wave (cm/sec)**		
	**CNTRL**	**PON**	**PON+siRNA-Not1**	**CNTRL**	**PON**	**PON+siRNA-Not1**
**Females**	42.15 ± 9.71	38.44 ± 16.98	44.62 ± 21.07	0.49 ± 0.13	0.89 ± 0.18	0.84 ± 0.90
**Males**	25.43 ± 3.51	29.78 ± 7.50	21.08 ± 1.63	1.77 ± 0.07	2.1 ± 0.54	2.30 ± 0.39
		**(b) cTn Levels**				
			**CNTRL**	**PON**	**PON+siRNA-Not1**	
		**Females**	0.0026 ± 0.002	0.0182 ± 0.007	0.009 ± 0.004	
		**Males**	0.0098 ± 0.006	0.039 ± 0.014	0.0212 ± 0.006	

LVEDD: left ventricular end-diastolic diameter; LVESD: left ventricular end-sistolic diameter; LVEF: left ventricular ejection fraction; cTn: cardiac troponin.

**Table 2:** Differentially activated and inhibited diseases and biofunctions.

Diseases and Biofunctions	Females	Males
Cell viability	4.542	−0.052
Cell survival	4494	−0.015
Transport of molecule	3014	1.802
Organismal death	−5241	−1.208

Activated or inhibited processes, involving Cx43, in hearts of mice treated with ponatinib compared to controls. Table reports the predictive z-score (orange:activation. Z-score ≥ 2.00; blue: inhibition, z-score ≤ −2.00).

**Table 3:** Antibodies used in this study.

Protein Target	Species	Dilution (IF)	Dilution (WB)	Catalog Number and Source
Cx43	rabbit	1:200	1:200	NB1000-91717, Novus Biologicals, Abingdon, UK
pS368-Cx43	rabbit	1:50	1:200	sc-101660, Santa Cruz Biotechnology, Santa Cruz Biotechnology, Santa Cruz, CA, USA
PKCps	rabbit	1:100		2261S, Cell Signalling Technology, Beverly, MA, USA
Cx26	rabbit	1:100	1:200	NBP2-41304, Novus biologicals
rabbit IgG	donkey	1:250		A10042, Alexa Fluor® 568 ThermoFisher Scientific, Waltham, Massachusetts, USA
GAPDH	rabbit		1:1000	G9545, Sigma Aldrich
rabbit IgG	goat		1:2000	#170-6515, Bio-Rad

## Data Availability

Data available upon request.

## Supplementary Materials

The following are available online at https://www.mdpi.com/article/10.3390/ijms22115815/s1.
